# Small Protease Sensitive Oligomers of PrP^Sc^ in Distinct Human Prions Determine Conversion Rate of PrP^C^

**DOI:** 10.1371/journal.ppat.1002835

**Published:** 2012-08-02

**Authors:** Chae Kim, Tracy Haldiman, Krystyna Surewicz, Yvonne Cohen, Wei Chen, Janis Blevins, Man-Sun Sy, Mark Cohen, Qingzhong Kong, Glenn C. Telling, Witold K. Surewicz, Jiri G. Safar

**Affiliations:** 1 National Prion Disease Surveillance Center, School of Medicine, Case Western Reserve University, Cleveland, Ohio, United States of America; 2 Department of Pathology, School of Medicine, Case Western Reserve University, Cleveland, Ohio, United States of America; 3 Department of Physiology and Biophysics, School of Medicine, Case Western Reserve University, Cleveland, Ohio, United States of America; 4 Department of Microbiology, Immunology and Pathology, Colorado State University, Fort Collins, Colorado, United States of America; Dartmouth Medical School, United States of America

## Abstract

The mammalian prions replicate by converting cellular prion protein (PrP^C^) into pathogenic conformational isoform (PrP^Sc^). Variations in prions, which cause different disease phenotypes, are referred to as strains. The mechanism of high-fidelity replication of prion strains in the absence of nucleic acid remains unsolved. We investigated the impact of different conformational characteristics of PrP^Sc^ on conversion of PrP^C^ in vitro using PrP^Sc^ seeds from the most frequent human prion disease worldwide, the Creutzfeldt-Jakob disease (sCJD). The conversion potency of a broad spectrum of distinct sCJD prions was governed by the level, conformation, and stability of small oligomers of the protease-sensitive (s) PrP^Sc^. The smallest most potent prions present in sCJD brains were composed only of∼20 monomers of PrP^Sc^. The tight correlation between conversion potency of small oligomers of human sPrP^Sc^ observed in vitro and duration of the disease suggests that sPrP^Sc^ conformers are an important determinant of prion strain characteristics that control the progression rate of the disease.

## Author Summary

Mammalian prion diseases were originally characterized by accumulation of protease-resistant prion protein (PrP^Sc^), often forming large amyloid deposits and fibrils. However, the apparent absence of protease-resistant PrP^Sc^ or amyloid fibrils in growing number of prion diseases raised several fundamental questions; specifically, whether presumably protease-sensitive forms of PrP^Sc^ exist as distinct conformers; and whether they comprise the initial steps in prion replication or are related to the alternative misfolding pathway generating noninfectious aggregates. We investigated the conformational characteristics of protease sensitive conformers of PrP^Sc^ and their role in the pathogenesis of sporadic Creutzfeldt-Jakob disease (sCJD). Using two different in vitro prion protein (PrP^C^) conversion techniques in tandem with biophysical methods, we identified small oligomers of protease sensitive PrP^Sc^ present in sCJD brains as the most potent initiators of PrP^C^ conversion. Their concentration and conformational stability determine the distinctly different replication potency of PrP^Sc^ in individual isolates of sCJD and each of these characteristics correlates tightly with duration of the disease. These features argue for a broad range of distinct prion strains causing the sCJD and imply that small oligomers of protease sensitive conformers of pathogenic prion protein are encoding incubation time and progression rate of the disease.

## Introduction

The yeast, fungal, and mammalian prions determine heritable and infectious traits, and thus behave like proteinaceous genes [1]. In mammals, prions cause a group of fatal and rapidly progressive neurodegenerative diseases, originally described as transmissible spongiform encephalopathies (TSEs) [1], [2]. The most common of the human prion diseases is sporadic Creutzfeldt-Jakob disease (sCJD) [3], accounting for ∼85% of all CJD cases worldwide [1]. Although the recent progress in the understanding of yeast and rodent-adapted prions was remarkable, whether or to what extent these findings can be applied to human prions is unclear [4] and the origin and pathogenesis of sCJD remains enigmatic [1]. Moreover, the number of prion strains that cause sCJD is not known [4]–[6] and, in contrast to growing structural characterization of rodent prions [7]–[9], no direct structural data are available for pathogenic prion protein (PrP^Sc^) present in sCJD brains beyond the evidence that it is variably resistant to proteolytic digestion [5], [10], [11].

Mammalian prion diseases were originally characterized by deposits of protease-resistant prion protein (PrP^Sc^), often forming large amyloid plaques and fibrils [12], [13]. Having a basic amino acid composition and an unstructured N-terminus, PrP can assume at least two conformations: native, α-helix-rich PrP^C^ [14] which is host-encoded by the chromosomal gene PRNP and expressed at different levels in mammalian cells [15]; and disease-causing, β-sheet-rich PrP^Sc^ [16], [17]. The prion hypothesis based on these findings posited that mammalian prions replicate by converting host's cellular prion protein (PrP^C^) into pathogenic protease resistant (r) conformational isoform (PrP^Sc^) [18]. However, the variable specific infectivity of rPrP^Sc^ and apparent absence of protease-resistant PrP^Sc^ or amyloid fibrils in growing number of prion diseases [11], [19] lead some researchers to question the causative link between rPrP^Sc^ and prion infectivity [6], [20], [21]. Apart from generating a controversy, these findings have raised fundamental questions; specifically, whether the amyloid or amyloid fibrils cause the disease; whether protease-sensitive (s) forms of PrP^Sc^ [22] comprise the initial steps in prion replication or are related to the alternative misfolding pathway generating noninfectious aggregates [4], [5]. Interestingly, subsequent experiments with purified and detergent-dissociated Syrian hamster PrP^Sc^ demonstrated a high seeding (replication) potency of small oligomers of the pathogenic prion protein [23]. Cumulatively, these findings raised an intriguing possibility that sPrP^Sc^ found invariably in sCJD-infected brains might be composed of such highly potent small oligomers.

Early important studies with mouse and Syrian hamster PrP^C^ demonstrated that the infectious PrP^Sc^ can be amplified indefinitely in crude brain homogenates by using alternating rounds of sonication and incubation, a procedure called serial protein misfolding cyclic amplification (sPMCA) [24]. Whether rPrP^Sc^ generated in PMCA is as infectious as the original brain derived sample is currently debated [25] but subsequent experiments with rodent, ungulate, and human prions proved that the procedure faithfully replicates the qualitative characteristics of various prion isolates [26]. In a parallel development, purified bacterially expressed recombinant (rec) PrP was shown to be converted by infectious rPrP^Sc^ in sonication-driven or quaking-induced conversion (QuIC), and yielded protease-resistant aggregates with a PK digestion pattern closely related to original brain PrP^Sc^ [27], [28]. Despite the low infectivity of recombinant replicas of Syrian hamster PrP^Sc^, these approaches helped to define some key elements of prion structure [27]–[29] and have shown specific and quantitative response to the brain-derived PrP^Sc^ used as a seed [30]–[32].

Although the PMCA and analogous techniques allowed to create prions “*de novo*” from recombinant proteins and thus prove in principle that mammalian prions are misfolded proteins [33]–[35], the remarkably precise mechanism replicating conformational features of PrP^Sc^ and translating them into unique phenotypes of the disease in different prion strains is largely unknown. To analyze the mechanistic and structural aspects of the replication of different sCJD prions and specifically the role of sPrP^Sc^, we employed an *in-vitro* amplification of brain PrP^Sc^ with QuIC and sPMCA. Using recombinant human PrP(23-231,129M) substrate in QuIC, and Tg mice brains expressing human PrP^C^(129M) in the sonication-driven sPMCA, both reactions demonstrated inverse correlation between conversion efficacy and the conformational stability of small protease sensitive (s) oligomers of PrP^Sc^. The observed link between duration of the disease and conversion potency of small oligomers of sPrP^Sc^ in individual sCJD cases suggests that these conformers encode the progression rate of the disease in different prion strains.

## Results

### Amplification index of sCJD PrP^Sc^ in sPMCA and QuIC

After four rounds of sPMCA, the Tg(HuPrP,129M) brain homogenate substrate responded to 10^6^-fold diluted sCJD seeds with matching codon 129 polymorphism and Type 1 or Type 2 PrP^Sc^ with levels of rPrP^Sc^ (PrP 27-30) easily detectable on WBs (**Fig. S1 A**). The WB mobility of unglycosylated fragments of rPrP^Sc^ from individual cases of sCJD matched the original Type 1 or Type 2 seeds (**Fig. S1 A**). The results extend previous observations on high fidelity replication of sCJD PrP^Sc^ using Tg mouse brain expressing homologous human PrP^C^ [26]. Whether subtle changes in glycosylation pattern also observed previously reflect the differences in the glycosylation of PrP^C^ in transgenic mice remains to be established [25], [26]. The PK treatment of rhuPrP^QuIC^ generated in QuIC resulted in a major PK-resistant fragment with SDS PAGE mobility corresponding to the mass ∼16 kDa (**Fig. S1 B**). We did not observe differences between masses of peptides generated from rhuPrP^QuIC^ seeded with Type 1 PrP^Sc^(129M) or Type 2 PrP^Sc^(129M). As expected [27], western blot indicated that the 16 kDa fragments, displaying all the epitopes downstream from residue 89, therefore correspond to the sequence ∼89–231 (**Fig. S1 B**).

**Figure 1:**
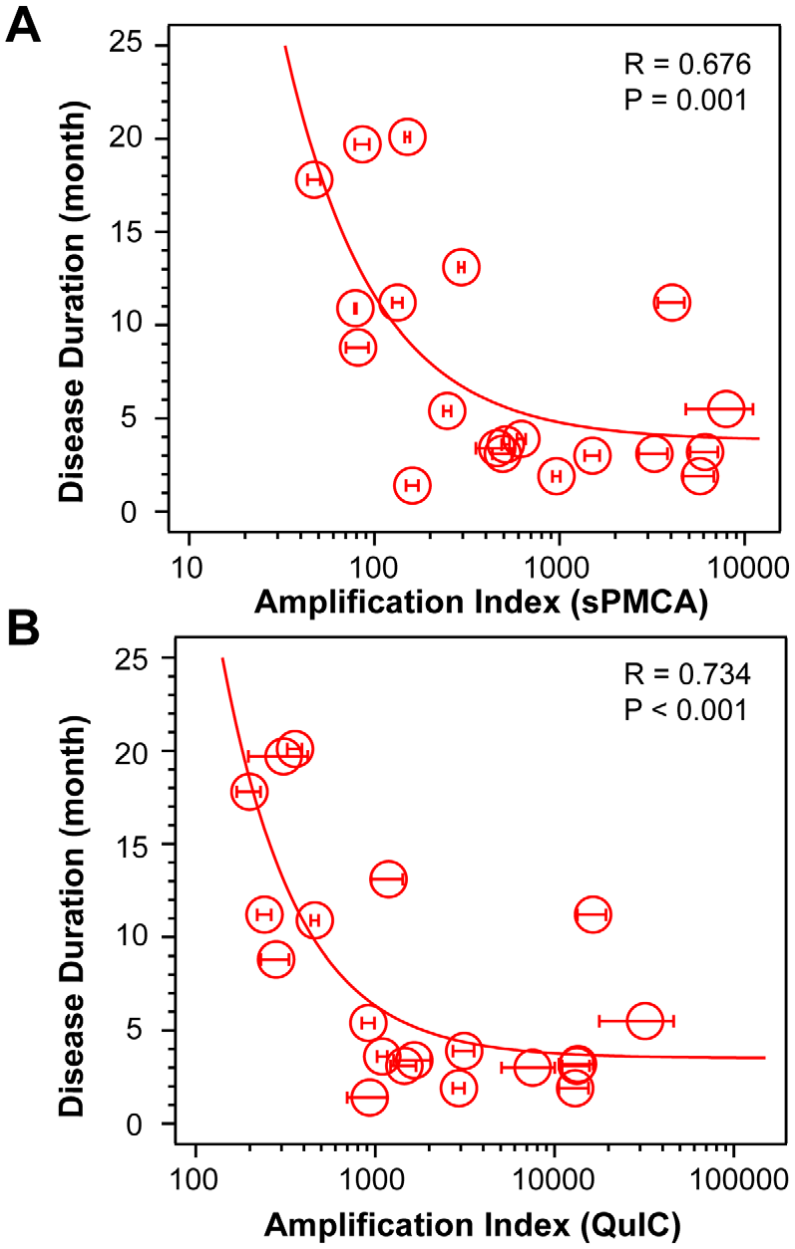
Conversion potency of sCJD PrP^Sc^ inversely mirrors duration of the sCJD. Amplification index obtained with (**A**) sPMCA or with (**B**) QuIC for MM1 (n = 10) and MM2 (n = 10) sCJD cases. The amplification index is the ratio between the concentration of PrP^Sc^ before and after sPMCA or QuIC measured with CDI. The data points and bars are averages ± SEM obtained from three independent conversion experiments, each measured in triplicate with CDI.

Using CDI, we measured the conversion potency of PrP^Sc^ present in sCJD brain homogenates of 20 patients homozygous for methionine in codon 129 and containing either Type 1 (n = 10) or Type 2 (n = 10) PrP^Sc^. Both techniques showed statistically significant inverse correlation of the amplification potency and duration of the sCJD (**Fig. 1A** **and** **Fig. 1B**). The CDI measurements were performed before and after four rounds of sPMCA (**Fig. 1A**) or after 20 hrs of QuIC (**Fig. 1B**), at the time points and dilutions that demonstrated maximum differences in amplification between different samples before they reached a plateau [36]. The amplification index (potency) of different seeds is expressed as a ratio between the concentration of the PrP^Sc^ conformers produced with PMCA divided by the concentration of PrP^Sc^ in the seed. In each run, the low background levels of PK-resistant PrP generated “*de novo*” in both sPMCA and QuIC in unseeded samples were subtracted from readings obtained with seeded samples (**Fig. S1A and Fig. S2A**). The tight correlation between amplification indexes obtained with sPMCA and QuIC in individual samples (**Fig. S2B**) proved that the observed range of values is highly reproducible with two different techniques. When we separated sCJD samples according WB type of PrP^Sc^, we observed overall higher seeding efficacy of MM1 PrP^Sc^ over MM2 PrP^Sc^ (**Fig. S3**). However, the trends were not statistically significant due to the broad range and overlapping amplification values. We concluded from these data that PrP^Sc^ present in cases classified as MM1 and MM2 sCJD display a continuum of conversion rates.

### The effect of protease treatment of the seeds on amplification rates

**Figure 2:**
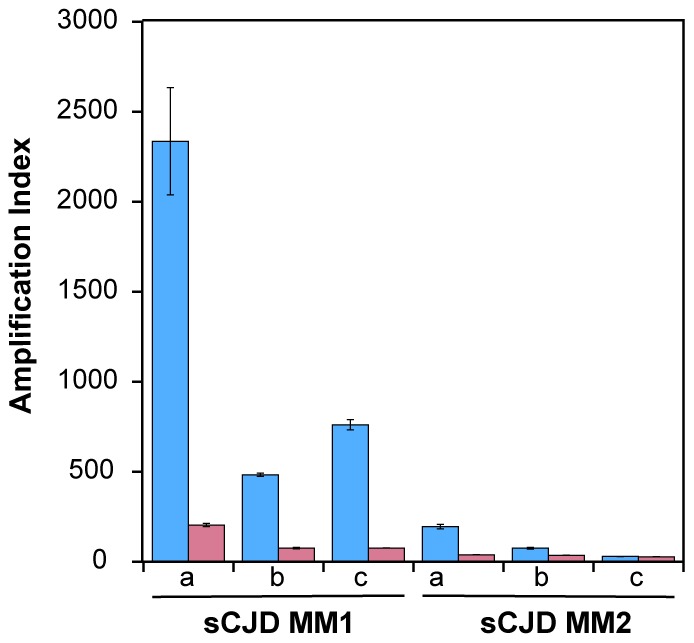
Decreased conversion potency of PrP^Sc^ after protease treatment. The sCJD brain homogenates were either (blue bars) untreated or (red bars) treated with 100 µg/ml of PK for 1 hr at 37°C. The PK was blocked with 0.5 mM PMSF, and aprotinin and leupeptin at 5 ug/ml, respectively. Impact of protease treatment of PrP^Sc^ on amplification was monitored in QuIC with CDI either in MM1 (n = 3) or MM2 (n = 3) sCJD cases. The bars represent average ± SEM from three QuIC experiments, each measured in duplicate with CDI.

Using seeds of Type 1 or Type 2 sCJD PrP^Sc^ that were either treated or not with PK before QuIC, we observed up to 10-fold higher seeding potency of the samples that were not treated with PK (**Fig. 2**). Despite the differences in the response of individual samples, this trend was most prominent with MM1 sCJD PrP^Sc^. With the CDI measurements of recombinant PrP before and after QuIC, we ruled out the possibility that incompletely inactivated PK decreased the concentration of the substrate (**Fig. S4**). We concluded from these experiments that in QuIC reaction, the total PrP^Sc^ is more efficient seed than protease-resistant fragment rPrP^Sc^.

### Conformational heterogeneity of MM1 and MM2 sCJD PrP^Sc^

The broad range of amplification indexes within each Type 1 or Type 2 group (**Fig. 1** **, Fig. S3 A, and Fig. S3 B**) suggests conformational heterogeneity beyond that observed with WBs. Therefore, we used the CDI [5], [22], [37] to determine the strain-dependent conformational range of sCJD PrP^Sc^ in patients who were homozygous for methionine in codon 129 of the PRNP gene and demonstrate pure Type 1 or 2 PrP^Sc^ on WBs [4]. The definition of sPrP^Sc^ as well as rPrP^Sc^ is operational and therefore all digestions with proteinase K (PK) were performed at constant protein/enzyme ratio equivalent to 3 IU/ml (∼100 µg/ml) in 10% brain homogenate containing 1% Sarkosyl for one hour at 37°C. The protocol for PrP^Sc^ digestion, validated in previously published experiments, was selected according to the following criteria: 1) complete digestion of PrP^C^ determined with CDI in control samples; 2) complete shift of the bands of PrP^Sc^ to PrP 27-30 on WBs; 3) unequivocal WB differentiation of Type 1 and Type 2 rPrP^Sc^ in all tested samples [4], [38]. The complete digestion of the PrP^Sc^ N-terminus with PK was monitored on WBs in all samples.

**Figure 3:**
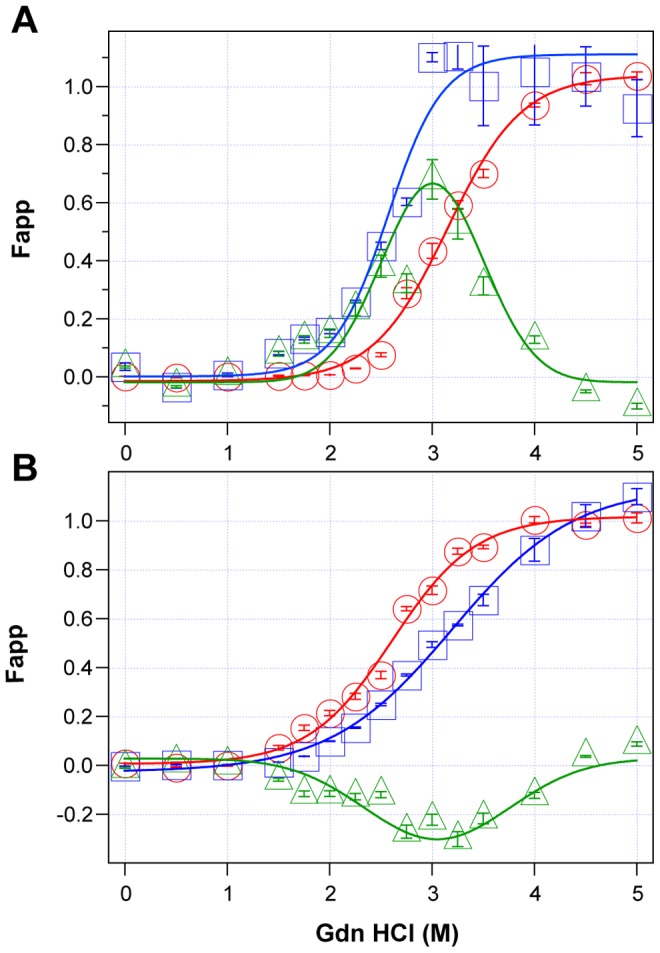
Impact of protease treatment on stability of PrP^Sc^ monitored with CDI. Typical dissociation and unfolding of (**A**) Type 1 PrP^Sc^(129M) and (**B**) Type 2 PrP^Sc^(129M) followed by CDI before (blue squares) and after (red circles) PK treatment; the differences in Fapp values before and after PK treatments are in triangles (green). The curves are the best fit with sigmoidal transition model to determine the midpoint (GdnHCl_1/2_ value) of the curve [4]. The differential values are fitted with the Gaussian model and the peak maximum corresponds to the mean stability of sPrP^Sc^ as described previously [4]. The values of apparent fractional change (Fapp) of each sample aliquot are mean ± SEM obtained from triplicate measurements.

**Figure 4:**
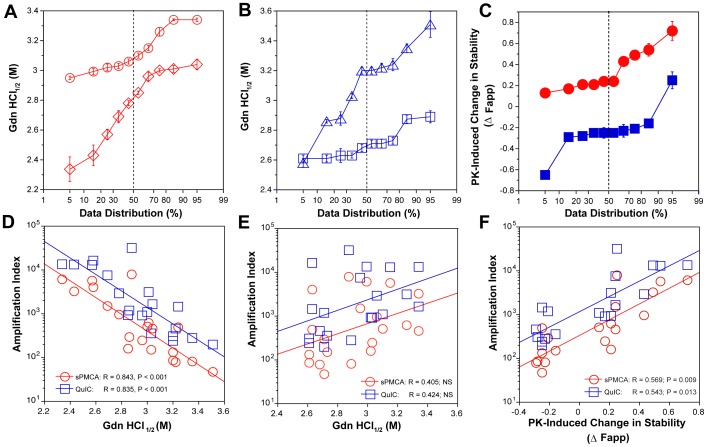
Relationship between conversion potency and the conformational stability of PrP^Sc^, rPrP^Sc^, and sPrP^Sc^ in MM1 (n = 10) and MM2 (n = 10) sCJD cases. The (**A**) conformational stability of MM1 PrP^Sc^ before (red diamonds) and after (red circles) PK digestion; the (**B**) conformational stability of MM2 PrP^Sc^ before (blue triangles) and after (blue squares) PK digestion; and the (**C**) fractional change in stability of PrP^Sc^ conformers induced by PK in individual sCJD samples (filled red circles) Type 1 PrP^Sc^(129M), and (filled blue squares) Type 2 PrP^Sc^(129M). The (**D**) inverse relationship between stability of total PrP^Sc^ and amplification index; (**E**) no correlation between stability of rPrP^Sc^ and amplification index; and (**F**) direct correlation between PK-induced change in the stability of PrP^Sc^ (Δ Fapp) and amplification index. The stability of prion and conversion potency of PrP^Sc^ was determined by CDI and expressed as Gdn HCl_1/2_ or stability change (Δ Fapp) induced by PK. Each symbol represents an average of triplicate experiment followed by triplicate measurement ± SEM with CDI.

Using CDI, we measured the concentration and conformational stability of PrP^Sc^ in the frontal cortex of individual sCJD patients used in the previous seeding experiments (**Table S1**). Typical examples of dissociation/unfolding curves before and after PK for MM1 and MM2 PrP^Sc^ are shown in **Fig. 3A and 3B**, respectively. Comparing ten sCJD Type 1 cases, we found a broad range of Gdn HCl_1/2_ values, from ∼2.3 to ∼3.0 M (**Table S1 and** **Fig. 4A**). We next investigated the conformational impact of the proteolytic digestion of sPrP^Sc^ conformers and the loss of N-terminal residues in rPrP^Sc^. The proteolysis of Type 1 PrP^Sc^(129M) with PK resulted in rPrP^Sc^ with invariably increased conformational stability (**Fig. 4A**).

Comparing ten sCJD Type 2 cases, we found a broad range of Gdn HCl_1/2_ values, from ∼2.6 to ∼3.5 M (**Table S1 and** **Fig. 4A**). In contrast to Type 1 sCJD PrP^Sc^, PK treatment of Type 2 PrP^Sc^(129M) uniformly, with one exception, produced rPrP^Sc^ with decreased stability (**Fig. 4b**). The unique case that showed the opposite trend displayed an atypical doublet of 19 and 17 kDa bands on WBs. Taken together, the data demonstrate wide range of unique conformations in both MM1 and MM2 subgroups. The Type 1 rPrP^Sc^(129M) remaining after proteolytic treatment demonstrated higher overall conformational stability than total PrP^Sc^. We observed the opposite effect of PK leading to less stable Type 2 rPrP^Sc^.

To quantify the impact of proteolytic treatment on the conformational stability of PrP^Sc^, we first evaluated the shift in the [Gdn HCl]_1/2_ values (**Table S1**). Alternatively, we subtracted the relative fractional change in stability of rPrP^Sc^ after PK treatment from the PrP^Sc^ values obtained before PK (**Table S1**, **Fig. 3** **, and** **Fig. 4C**). The resulting differential curves corresponding to the portion of PrP^Sc^ conformers removed by PK and designated operationally sPrP^Sc^ [4]. The differential curves exhibit Gaussian distribution with the peak at the median stability of sPrP^Sc^; the height and integrated peak area is proportional to the relative fraction of PK-digested conformers (ΔFapp). In contrast to simple shift in the [Gdn HCl]_1/2_ values, the ΔFapp take into account the difference in the slopes of the unfolding curves. Using both shifts in the [Gdn HCl]_1/2_ and ΔFapp values, the overall stability of Type 1 sPrP^Sc^ is lower than that of rPrP^Sc^ (**Fig. 4C**). In contrast, the negative differential curves for Type 2 sPrP^Sc^(129M) and shift in the [Gdn HCl]_1/2_ values induced by PK both demonstrate that sPrP^Sc^ is more stable than rPrP^Sc^ in this sCJD group (**Fig. 4C**). Cumulatively, the data provide important evidence that MM1 and MM2 PrP^Sc^ conformers differ in response to proteolytic cleavage, but the observed spread of stability values within each sCJD WB pattern suggests the presence of a broad range of unique PrP^Sc^ conformers. Alternatively, data might be also consistent with varying ratios of a small set of conformers that, in themselves, are not unique to each sCJD patient [4], [39].

### The amplification rates inversely correlate with the stability of sCJD sPrP^Sc^

Comparison of the stability of total PrP^Sc^ with the amplification efficacy of individual sCJD PrP^Sc^ seeds demonstrate a highly significant inverse correlation for both MM1 (n = 10) and MM2 (n = 10) sCJD samples, and in both sPMCA and QuIC (**Fig. 4D**). Since this correlation was lost after PK treatment (**Fig. 4E**) we concluded that sPrP^Sc^ fraction of PrP^Sc^ must be responsible for the initial correlation. In confirmation, the change in the stability induced by PK correlated to a highly significant degree with the amplification rate (**Table S1** and **Fig. 4F**). Specifically, the samples with less stable sPrP^Sc^ (positive shift in [Gdn HCl]_1/2_ or in Fapp value) showed higher amplification rates (**Fig. 4F**), and vice versa. We concluded from these experiments that the amplification rates of sCJD PrP^Sc^ correlate inversely with the stability of total PrP^Sc^, and that less stable sPrP^Sc^ conformers are responsible for this effect. In contrast, the stability of the rPrP^Sc^ conformers does not predict the amplification rate of individual sCJD samples, regardless of the WB type.

### The relationship between prion size, protease sensitivity, and conversion efficacy of sCJD PrP^Sc^

**Figure 5:**
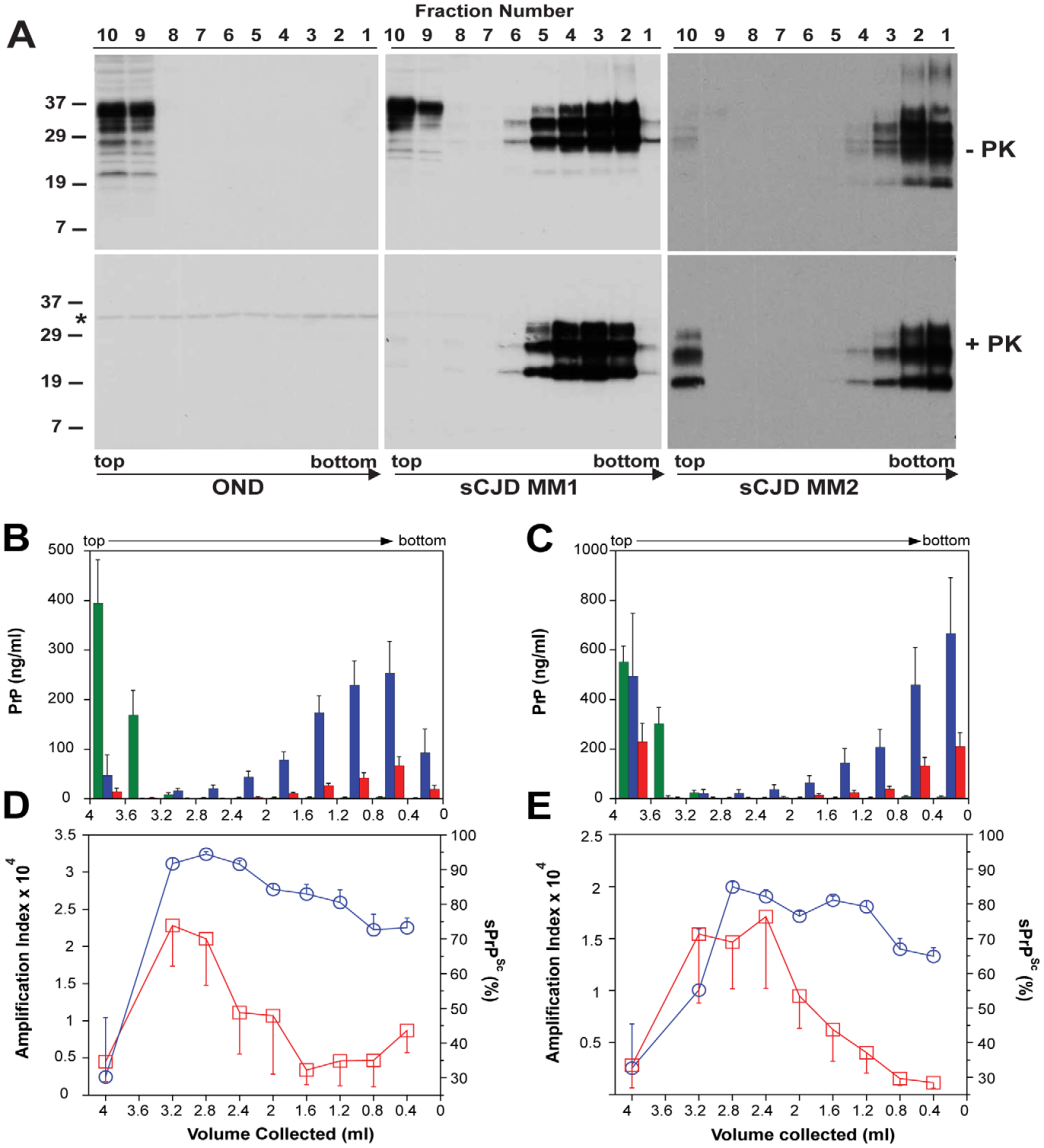
Sedimentation velocity, amplification index, and protease sensitivity of PrP^Sc^ present in frontal cortex of patients with sCJD Type MM1 (n = 6) and Type MM2 (n = 6) and fractionated by ultracentrifugation in sucrose gradient. Fractions were collected from the bottom of the tubes and PK-treated or untreated samples were analyzed for PrP by (**A**) WB with biotinylated primary antibody 3F4 and secondary Streptavidin-Peroxidase complex. The WBs are typical examples of Other Neurological Disease (OND), Type 1 (sCJD MM1), and Type 2 sCJD (sCJD MM2) homozygous for methionine in codon 129 of PRNP gene. The cumulative plots of concentration of PrP^C^ (green bars), total PrP^Sc^ (blue bars), and rPrP^Sc^ (red bars) in sucrose fractions was determined with CDI before or after PK treatment in six cases of (**B**) sCJD type MM1 and six cases of (**C**) Type MM2. The bars represent average ± SEM (n = 6); CDI was performed in each sCJD case in triplicate. The amplification index (red squares) determined with QuIC and relative concentration of sPrP^Sc^ (blue circles) measured by CDI and QuIC in (**D**) MM1 (n = 6) and (**E**) MM2 (n = 6) sCJD PrP^Sc^ separated in sucrose gradient. The points and bars are average ± SEM; both QuIC and CDI were performed in triplicate for each sCJD case sample. The asterisk in WBs indicates the band of PK cross reacting with primary antibody. The molecular mass of the markers is in kDa.

To investigate the impact of prion particle size on protease sensitivity and amplification, we separated sCJD prion particles according to sedimentation velocity using high-speed centrifugation in sucrose gradient. The sCJD prions present in brain homogenates of twelve sCJD patients with MM1 (n = 6) or MM2 (n = 6) PRNP gene polymorphism and WB pattern of PrPSc were separated in 10–45% sucrose gradient and collected fractions were analyzed by WBs, CDI, and QuIC. The PrP^C^ in platelets of healthy donors as well as in brain tissue of patients that had other neurologic disorders remained in the top portion of the tube, as expected for monomers or possible dimers of PrP^C^ (**Fig. S6**). Similarly, PrP^C^ present in MM1 and MM2 sCJD cases remained in the upper portions of the tubes (**Fig. 5A** **,** **Fig. 5B** **, and** **Fig. 5C**). In contrast, the MM1 (n = 6) and MM2 (n = 6) PrP^Sc^ sedimented into sucrose with a broad range of densities, and a variable fraction remained flotating (**Fig. 5B** **and** **Fig. 5C**). The peak sedimentation velocity of MM1 PrP^Sc^ is reproducibly slower than that of MM2 PrP^Sc^ (**Fig. 5A** **,** **Fig. 5B** **, and** **Fig. 5C**). The distinctive sedimentation velocity of the majority of MM2 sCJD PrP^Sc^ conformers is remarkably reproducible and independent of total PrP^Sc^ concentration (**Fig. S7**). On the basis of calibration with standard proteins (**Fig. S5**) and sω^2^t value distribution in the sucrose gradient, we estimate that the range of S values of CJD prions is from ∼20 to >150. The majority of MM1 PrP^Sc^ conformers have an S value in the range 110–130. In contrast, the S value for the majority of MM2 PrP^Sc^ conformers is ≥150. We concluded that both MM1 PrP^Sc^ and MM2 PrP^Sc^ display a broad range of aggregates and that the MM1 PrP^Sc^ aggregates are reproducibly smaller that those composed of MM2 PrP^Sc^. The variable low-density flotating fraction of PrP^Sc^ in both MM1 and MM2 PrP^Sc^ samples suggests that the aggregates are very small, or that some PrP^Sc^ exists in a complex with detergent-insoluble low-density lipids [40]. We estimate that the most frequent aggregates in MM1 sCJD PrP^Sc^ have mass 9–11×10^6^ Da and are composed of ∼380–460 monomers of PrP^Sc^. The most frequently occurring aggregates in MM2 sCJD PrP^Sc^ have mass ≥14×10^6^ Da and are composed of ≥600 monomers of PrP^Sc^.

The amplification index obtained from each fraction with QuIC showed the highest conversion potency for the fractions with S values from 18 to 30 and significantly lower amplification potency of PrP^Sc^ with increasing size of the aggregates in MM1 (n = 6) as well as MM2 (n = 6) sCJD cases (**Fig. 5D** **and** **Fig. 5E**). The PK resistance profile of both MM1 and MM2 PrP^Sc^ obtained with CDI indicates a statistically significant (MM1: P<0.001; MM2: P = 0.034) trend toward higher PK sensitivity of the fractions containing small oligomers of PrP^Sc^ in comparison to the bottom and floating fractions (**Fig. 5D** **, and** **Fig. 5E**). However, this trend is difficult to evaluate on the WBs because the impact of PK is masked by a ∼3.5-fold higher affinity of mAb 3F4 to PK-cleaved PrPSc [4]. The same effect is responsible for an apparently higher signal of PrPSc observed after proteolytic treatment in the floating fraction of MM2 sCJD (**Fig. 5A**). In CDI, the impact of differential mAb binding is corrected by separate calibration with HuPrP(23-231) for PK-untreated samples and with HuPrP(90-231) for PK-treated samples [4].

**Figure 6:**
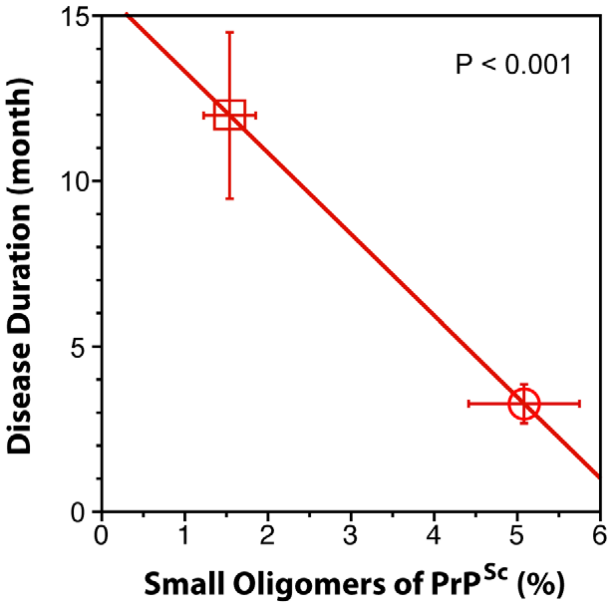
The inverse relationship between proportion of small oligomers of PrPSc and duration of sCJD. The levels of small oligomers of PrPSc with the highest seeding potency that eluted at 2.4–3.2 ml of sucrose gradient (**Fig. 5D and Fig. 5E**) were expressed as a percentage of total PrPSc in (circle) MM1 (n = 6) and (square) MM2 (n = 6) sCJD cases. Each symbol represents an average ± SEM.

When we expressed the levels of small oligomers of PrPSc with the highest seeding potency that eluted between 2.4–3.2 ml of sucrose gradient as a percentage of total PrPSc, we observed an inverse correlation with the duration of the sCJD (**Fig. 6**). Cumulatively, the diminished amplification potency of the sCJD prions after protease treatment, the stability and sedimentation velocity data indicate that the highest seeding potential in QuIC have relatively PK-sensitive small aggregates of PrP^Sc^. We estimate that the most potent seeds of sCJD PrP^Sc^ have a mass 0.45–1.80×10^6^ Da and are composed of 20–78 monomers of PrP^Sc^. The control experiments with standard proteins performed in the presence or absence of Sarkosyl indicate that Sarkosyl binding influenced sedimentation velocity by ∼8%. Additional error in our estimates could come from lipids bound in PrP^Sc^ aggregates. However, assuming a 2∶1 molar ratio of PrP^Sc^ monomer to sphingolipids and cholesterol remaining in PrP^Sc^ aggregates after detergent solubilization and centrifugation in sucrose gradient [41], the error of our estimates due to the lipid content does not exceed 4%.

## Discussion

In this study, we used novel conformational methods derived from CDI in tandem with two different amplification techniques for PrP^Sc^ to determine the spectrum of sCJD prions and to investigate conformational features that control conversion reaction. Surprisingly, we identified within each clinical and pathologic category of sCJD an array of PrP^Sc^ structures that differ in protease-sensitivity, conformational stability, and conversion potency. Each of these features offers evidence of a distinct prion strain and suggests that the extraordinary clinicopathologic variability of sCJD stems from the broad range of prions causing the disease and imply stochastic origin or conformational evolution during prion propagation in the brain. Our data on human brain sCJD prions indicate an intimate relationship between the conformation, size, and conversion potency of the small protease-sensitive oligomers of PrP^Sc^.

### Sources of phenotypic and molecular diversity of sCJD

The distinct conversion efficacies we observed with prion seeds obtained from different cases of MM1 and MM2 sCJD were highly reproducible with both QuIC and sPMCA techniques (**Fig. S2**). Cumulatively, the data demonstrate that the conversion potency of PrPSc in individual isolates of sCJD is inversely related to the duration of the disease. Because the length of the duration of a clinically pronounced prion disease is a function of the incubation time, this finding accords with the bioassay data that show a broad spectrum of transmission rates and incubation times of sCJD prions in transgenic mice expressing human PrP^C^(129M) or human/mouse PrP^C^ chimeras [5], [6], [42]. Therefore, our demonstration that disease duration in sCJD patients mirrored the conversion efficacy of PrPSc suggests that both sPMCA and QuIC reproduce important kinetic aspects of prion replication in vivo, though infectivity properties of both reaction products are yet to be tested. The distinctness in replication efficacy is a principal hallmark of prion strains; it leads to different incubation times and progression rates of the disease in bioassays [1]. The statistically insignificant difference between Type MM1 PrP^Sc^ and MM2 PrP^Sc^ and broad distribution of values within each group suggests that a continuum of sCJD prions are causing the disease.

The striking range of conformational stability values of PrP^Sc^ found with CDI before or after PK in sCJD patients homozygous for codon 129 plymorphism of the PRNP gene and showing either pure Type 1 or Type 2 WB pattern (**Fig. 4A** **and** **Fig. 4B**) by far exceeds the variability expected from the sampling or method itself [4]. The observed differences in stability offer evidence of a broad spectrum of different conformations of PrP^Sc^ present in vivo in MM1 and in MM2 sCJD patients [4], [43]. The increased frequency of exposed epitopes and decreased stability in Type 2 PrPSc after PK treatment [4] are counterintuitive and may indicate one of three possibilities: that the PK sensitivity is not an obligatory measure of protein stability and rPrPSc may be in some prion strains less stable than sPrPSc; that removal of the N-terminus from PrPSc resulted in less stable conformation with more exposed 108–112 epitopes; or that the ligand protecting the 108–112 epitope and stabilizing the PrPSc was removed by PK. Whether the epitopes' hindrance in undigested PrPSc is the result of lipid, glycosaminoglycan, nucleic acid, or protein binding to the conformers unique to the MM2 sCJD PrPSc remains to be established. Since sCJD cases with Type 2 PrPSc(129M) have generally extended disease durations, the molecular mechanism underlying this effect calls for detailed investigation. Cumulatively, the conversion and stability data support the conclusion that each MM1 or MM2 category of sCJD prions causes an accumulation of a broad range of distinct conformers of PrP^Sc^ in the individual sCJD brains. Whether individual sCJD case is caused by a single conformer having unique replication efficacy or a varying ratios of a small set of conformers that, in themselves, are not unique to each sCJD patient remains to be seen. Consequently, since all these phenomena characterize prion strains, the data suggest that a broad range of prion strains exists within each clinicopathologic category of sCJD.

### The impact of levels and stability of protease-sensitive conformers of PrP^Sc^ on conversion potency of human prions

Considerable data demonstrate that sPrP^Sc^ replicates in vivo and in vitro as an invariant and major fraction of PrP^Sc^ and that the proteolytic sensitivity of PrP^Sc^ can reliably differentiate various prion strains [5], [22], [44], [45]. Accumulation of sPrP^Sc^ precedes protease-resistant product (rPrP^Sc^) in prion infection [46]; and up to 90% of PrP^Sc^ accumulating in CJD brains consists of sPrP^Sc^ [5], [47]. It is noteworthy that amyloid fibrils produced in vitro from recombinant mouse PrP generated prions composed exclusively of sPrP^Sc^ upon inoculation into wild mice [19].

Previously we found that levels of sPrP^Sc^ varied with the incubation time of the disease [22] and we hypothesized that the molecular mechanism responsible for this phenomenon was related to the replication or clearance of prions [5], [22]. Subsequent experiments in yeast indicated that replication rate of prions may be an inverse function of the stability of misfolded protein [48]. The hypothesis based on these experiments posits that the less stable prions replicate faster by exposing more available sites for growth of the aggregates. Although more recent experiments with rodent and synthetic prions showed the correlation between shorter incubation time of prions that converted to protease-sensitive isoforms at a lower denaturant concentrations. the replication rate of mammalian prions was never measured and the observed effect could be equally well explained by different clearance rates of distinct prions [49], [50].

The experiments presented here determined the impact of the overall stability of sCJD PrP^Sc^, rPrP^Sc^, and sPrP^Sc^ on the seeding potency of sCJD prions with QuIC and sPMCA. Both methods revealed that the stability of total PrP^Sc^ inversely correlated with the amplification index of sCJD PrP^Sc^. Moreover, when sPrP^Sc^ proved less stable than rPrP^Sc^, the difference in stability correlated with more efficient amplification. Conversely, when sPrP conformers proved more stable than rPrP^Sc^, we observed the opposite effect-less accumulated PrP^Sc^ in both sPMCA and QuIC (**Fig. 4**). The absent clearance in both methods lead to the conclusion that lower stability of PrP^Sc^ conformers, and specifically sPrP^Sc^ conformers, is an important determinant of the conversion rate and that these conformers likely play an important role in the incubation time and progression of the diseases in vivo.

### The relationship between conversion efficacy and prion size

Although prion diseases were originally characterized by deposits of protease-resistant PrP^Sc^, often with large amyloid fibrils, there is growing recognition that protease resistance and amyloid fibrils do not constitute obligatory factors in the pathogenesis of these diseases [5], [11], [22]. These findings have accordingly raised certain questions; specifically, whether the amyloid fibrils cause the disease; and whether smaller non-amyloid oligomers of PrP^Sc^ comprise the initial steps in prion replication, or are related to the alternative misfolding pathway [5], [22], [44]. Subsequent evaluation of the converting activity and of the size of aggregates dissociated from purified Syrian hamster PrP^Sc^ with ionic detergent sodium n-undecyl sulphate suggested that the maximum seeding activity is associated with relatively small aggregates dissociated from longer fibrils [23]. However, it remained unclear whether such highly potent small oligomers exist also in vivo.

In this research, we investigated whether small aggregates of PrP^Sc^ with high seeding potency exist in the brains of patients with sCJD. The broad range of sedimentation velocities observed with ultracentrifugation of sCJD brain homogenates in sucrose gradient indicate that sCJD PrP^Sc^ proteins exist in the continuum of aggregates composed from <20 to >600 PrP^Sc^ molecules. Surprisingly, small oligomers of human PrP^Sc^, with masses equivalent to 20-78 PrP^Sc^ molecules, are the most efficient initiators of PrP^C^ conversion, and the seeding efficacy of sCJD prions actually decreased with the size of the aggregates. Interestingly, the variable fraction of PrP^Sc^ remained with PrP^C^ in the upper layers of sucrose gradient. This phenomenon may suggest even smaller oligomers or, alternatively, association with detergent-insoluble lipids present in cholesterol-rich domains (caveolae) of cellular membranes. This association would place both cellular and pathogenic forms of the prion protein into the same compartment and thus support the hypothesis that PrP^Sc^ formation occurs within caveolae [40].

The strikingly high sedimentation velocity of MM2 PrP^Sc^, in contrast with the lower sedimentation velocity of MM1 PrP^Sc^ indicates that MM2 PrP^Sc^ forms much larger aggregates and concurs with the pattern of large coarse deposits of Type 2 PrP^Sc^ observed with the brain immunohistochemistry in situ, in contrast to the fine (punctate, “synaptic”) appearance of the immunoreactivity associated with Type 1 PrP^Sc^ [10]. Cumulatively, these data suggest that the distinct quaternary structure or packing of the monomers of PrP^Sc^ may be responsible for the different peptide fragmentation pattern with predominantly 19 kDa fragments of MM2 rPrP^Sc^ and 21 kDa in MM1 rPrP^Sc^ after PK treatment.

## Materials and Methods

### Ethics statement

All procedures were performed under protocols approved by the Institutional Review Board at Case Western Reserve University. In all cases, written informed consent for research was obtained from patients or legal guardians and the material used had appropriate ethical approval for use in this project. All patients' data and samples were coded and handled according to NIH guidelines to protect patients' identities.

### Patients and clinical evaluations

We selected 20 representative subjects from a group of 340 patients with definitive diagnosis of sCJD. The criteria for inclusion were (1) availability of clinical diagnosis of CJD according to WHO criteria [51], [52] and clearly determined and dated initial symptoms upon neurologic examination to ascertain the disease duration; (2) methionine homozygous at codon 129 of the human prion protein (PrP) gene (PRNP); (3) unequivocal classification as pure Type 1 or Type 2 sCJD according to WB pattern; (4) unequivocal classification of pathology as definite Type 1 or 2 at the National Prion Disease Pathology Surveillance Center (NPDPSC) in Cleveland, Ohio; (5) demographic data distribution within 95% confidence interval of the whole group, resulting in no difference between selected cases and the whole group in any of the statistically followed parameters.

Retrospective charts review was carried out for all subjects, with particular attention to the documented initial cardinal clinical signs of sCJD such as cognitive impairment, ataxia, and myoclonus [51], [52]. We also reviewed the findings on electroencephalography, brain magnetic resonance imaging, and CSF markers when available.

### Brain samples and PRNP gene sequencing

All Type 1–2 patients or uncertain cases were excluded from this study. DNA was extracted from frozen brain tissues in all cases, and genotypic analysis of the PRNP coding region was performed as described [5], . On the basis of diagnostic pathology, immunohistochemisty, and western blot (WB) examination of 2 or 3 brain regions (including frontal, occipital and cerebellum cortices) with mAb 3F4, the pathogenic PrP^Sc^ was classified as (1) Type 1 PrP^Sc^(129M) (n = 10) and (2) Type 2 PrP^Sc^ (129M, n = 10). Patients lacked pathogenic mutations in the PRNP and had no history of familial diseases or known exposure to prion agents. These cases underwent additional detailed WB analyses of the PrP^Sc^ so that we could ascertain the accuracy of their original classification and confirm that the same brain homogenate analyzed by CDI contained pure Type 1 PrP^Sc^(129M) or Type 2 PrP^Sc^(129M).

Coronal sections of human brain tissues were obtained at autopsy and stored at −80°C. Three 200–350 mg cuts of frontal (superior and more posterior middle gyri) cortex were taken from each brain and used for molecular analyses. The other symmetric cerebral hemisphere was fixed in formalin and used for histologic and immunohistochemical purposes.

### Brain homogenates and precipitation of prions with PTA

Slices of tissues weighing 200–350 mg were first homogenized to a final 15% (w/v) concentration in calcium-free and magnesium-free PBS, pH 7.4, by 3 75 s cycles with Mini-beadbeater 16 Cell Disrupter (Biospec, Bartlesville, Oklahoma). The homogenates were then diluted to a final 5% (w/v) in 1% (v/v) Sarkosyl in PBS, pH 7.4 and rehomogenized. After clarification at 500×g for 5 min, one aliquot of the supernatant was treated with protease inhibitors (0.5 mM PMSF and aprotinin and leupeptin at 5 ug/ml, respectively). The second aliquot was treated with 50 µg/ml of proteinase K (Amresco, Solon, Ohio) for 1 h at 37°C shaking 600 rpm on Eppendorf Thermomixer (Eppendorf, Hauppauge, New York) and PK was blocked with PMSF and aprotinin-leupeptin cocktail. Both aliquots were precipitated with final 0.32% (v/v) NaPTA after 1 h incubation at 37°C as described [22]. The samples were spun 30 min at 14,000×g in Allegra X-22R tabletop centrifuge (Beckman Coulter, Brea, California) and the pellets were resuspended in 250 ul of deionized water containing protease inhibitors (0.05 mM PMSF, aprotinin and leupeptin at 1 ug/ml each, respectively, and stored for analysis at −80°C.

### Western blots

Both PK-treated and untreated samples were diluted 9-fold in 1× Laemmli Buffer (Bio-Rad, Hercules, California) containing 5% (v/v) beta-mercaptoethanol (ME) and final 115 mM Tris-HCl, pH 6.8. Samples were heated for 5 min at 100°C and ∼2 ng of PrP per lane was loaded onto 1 mm 15% Polyacrylamide Tris-HCl, SDS-PAGE gels (Bio-Rad) mounted in Bio-Rad Western Blot apparatus. After electro-transfer to Immobilon-P Transfer Membranes (Millipore, Bedford, Massachusetts), the membranes were blocked with 2% (w/v) BSA in TBS containing 0.1% of Tween 20 (v/v) and 0.05% (v/v) Kathon CG/ICP (Sigma, St. Louis, Missouri). The PVDF membranes were developed with 0.05 ug/ml of biotinylated mAb 3F4 (Covance, Princeton, New Jersey) followed by 0.0175 ug/ml Streptavidin-Peroxidase conjugate (Fisher Scientific, Pittsburgh, Pennsylvania) or with ascitic fluid containing mAb 3F4 (kindly supplied by Richard Kascsak) diluted 1∶20,000 followed by Peroxidase-labeled sheep anti-mouse IgG Ab (Amersham, Piscataway, New Jersey) and diluted 1∶3000. The membranes were developed with the ECL Plus detection system (Amersham) and exposed to Kodak BioMax MR Films (Fisher Scientific) or Kodak BioMax XAR Films (Fisher Scientific).

### Conformation-dependent immunoassay (CDI)

The CDI for human PrP was performed as described previously [4], [5], [54], with several modifications. First, we used white Lumitrac 600 High Binding Plates (E&K Scientific, Santa Clara, California) coated with mAb 8H4 (epitope 175–185) [55] in 200 mM NaH_2_PO_4_ containing 0.03% (w/v) NaN_3_, pH 7.5. Second, aliquots of 20 µl from each fraction containing 0.007% (v/v) of Patent Blue V (Sigma) were directly loaded into wells of white strip plates prefilled with 200 µl of Assay Buffer (Perkin Elmer, Waltham, Massachusetts). Finally, the captured PrP was detected by a europium-conjugated [22] anti-PrP mAb 3F4 (epitope 107–112) [56] and the time-resolved fluorescence (TRF) signals were measured by the multi-mode microplate reader PHERAstar Plus (BMG LabTech, Durham, North Carolina). The recHuPrP(90-231,129M) and PrP(23-231,129M) used as a calibrant in the CDI was prepared and purified as described previously [57]. The initial concentration of recombinant human PrP(23-231) and PreP(90-231) was calculated from absorbance at 280 nm and molar extinction coefficient 56650 M^−1^ cm^−1^ and 21640 M^−1^ cm^−1^, respectively. The purified recombinant proteins were dissolved in 4 M GdnHCl and 50% Stabilcoat (SurModics, Eden Prairie, Minnesota), and stored at −80°C. The concentration of PrP was calculated from the CDI signal of denatured samples using calibration cure prepared with either recPrP(23-231) for samples containing full length PrP^Sc^ or recPrP(90-231) for samples containing truncated rPrP^Sc^ (PrP 27-30) after proteinase-K treatment. This separate calibration was necessary due to the ∼3.5-fold lower affinity of mAb 3F4 with full-length human PrP(23-231,129M) compared to PrP(90-231,129M) [4].

### Monitoring dissociation and unfolding of PrP^Sc^ by CDI

The denaturation of human PrP^Sc^ was performed as described previously [4], [22], with several modifications. Frozen aliquots of PrP^Sc^ were thawed, sonicated 3×5 s at 60% power with Sonicator 4000 (Qsonica, Newtown, Connecticut), and the concentration was adjusted to constant ∼50 ng/ml of PrP^Sc^. The 15 µl aliquots in 15 tubes were treated with increasing concentrations of 8 M GdnHCl containing 0.007% (v/v) Patent Blue V (Sigma, St. Louis, Missouri) in 0.25 M or 0.5 M increments. After 30 min incubation at room temperature, individual samples were rapidly diluted with Assay Buffer (Perkin Elmer, Waltham, Massachusetts) containing diminishing concentrations of 8 M GdnHCl, so that the final concentration in all samples was 0.411 M. Each individual aliquot was immediately loaded in triplicate to dry white Lumitrac 600, High Binding Plates (E&K Scientific, Santa Clara, California), coated with mAb 8H4, and developed in accordance with CDI protocol using europium-labeled mAb 3F4 for detection [4], [5], [22], [37], [58].

The raw TRF signal was converted into the apparent fractional change of unfolding (Fapp) as follows [4]: F = (TRF_OBS_−TRF_N_)/(TRF_U_−TRF_N_) where TRF_OBS_ is the observed TRF value, and TRF_N_ and TRF_U_ are the TRF values for native and unfolded forms, respectively, at the given Gdn HCl concentration [17]. To determine the concentration of Gdn HCl where 50% of PrP^Sc^ is unfolded ([Gdn HCl]_1/2_), the data were fitted by least square method with a sigmoidal transition model (Equation 1):


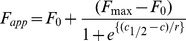


The apparent fractional change (F_app_) in the TRF signal is the function of Gdn HCl concentration(c); c_1/2_ is the concentration of Gdn HCl at which 50% of PrP^Sc^ is dissociated/unfolded and r is the slope constant. To determine the impact of protease treatment on the conformational stability of PrP^Sc^, the values of fractional change after PK were subtracted from F_app_ values obtained before PK (ΔF_app_ = F^0^−F^PK^) and then fitted with a Gaussian model to estimate the proportion and average stability of sPrP^Sc^ conformers (Equation 2):


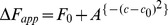


In this model, the Pk-induced fractional change is ΔF_app_, F_0_ is fractional change at 0 concentration of Gdn HCl, and c_0_ is the Gdn HCl concentration at the maximum height A of the peak [4].

### Quaking-Induced Conversion(QuIC)

The QuIC was performed as described [27] with the following modifications. The rhuPrP(90-231,129M) used as a substrate in QuIC was expressed, purified, and refolded to α-helical conformation as described previously [57]. The initial concentration of recombinant human PrP(23-231) was calculated from absorbance at 280 nm and molar extinction coefficient 56650 M^−1^ cm^−1^. The stock of rhuPrP(23-231) in 10 mM Sodium Acetate buffer, pH 4.0, was pretreated with 12 mM HCl at 1∶3.9 (rhuHuPrP ∶ HCl, v/v) ratio for 7.5 min and immediately diluted to final 0.1 mg/ml into the reaction buffer composed of 20 mM NaH_2_PO_4_, 130 mM NaCl, pH 6.9, and containing 0.1% SDS, 0.1% Triton X-100, and 1∶5000 (v/v) N2 (Invitrogen, Carlsbad, California). The QuIC was performed with final volume 100 µl per well in sterile V-bottom, low-binding polypropylene 96-well plate (VWR, Arlington Heights, Illinois) equipped with a 3 mm diameter PTFE bead (Fisher Scientific, Pittsburgh, Pennsylvania) in each well. The aliquots of sCJD brain homogenates were diluted into the complete QuIC reaction buffer to obtain final 10^−4^ dilution of sCJD prions, and the plates were sealed with sterile AxyMat Silicone Sealing Mat (VWR, Arlington Heights, Illinois). The QuIC was performed in samples seeded with sCJD PrP^Sc^ at 55°C for 20 hrs in Eppendorf Thermomixer (Eppendorf, Hauppauge, New York) set for 1 min shaking at 1400 rpm followed by 1 min incubation.

To each well containing 100 µl of QuIC reaction buffer was added 50 µl of PBS, pH 6.9, containing 3% (w/v) Sarkosyl and Proteinase K (PK; Amresco, Solon, Ohio) to obtain the final Sarkosyl concentration 1% (w/v) and PrP/enzyme ratio 10∶1 (w/w). The plates was incubated for 1 h at 37°C at 1200 rpm on the Eppendorf Thermomixer with 1 min interval. The PK was blocked in each well with protease inhibitors (0.5 mM PMSF final, and 5 ug/ml of aprotinin and leupeptin).

### Serial replication of prions in vitro by sonication-driven (s) PMCA

Sonication-driven PMCA of sCJD samples was performed as described [26] with the following modifications. Human PrP^Sc^ was replicated using brains of transgenic mice overexpressing human PrP with methionine at position 129 [59], [60]. The 10% brain homogenates from sCJD patients were diluted 1000-fold into 10% normal brain homogenate and 100 µl was transferred into 0.2 ml PCR tubes equipped with 2.38 mm diameter PTFE ball (K-mac Plastics, Wyoming, Michigan). Tubes were positioned on an adaptor placed on the plate holder of a microsonicator (Misonix Model 3000, Farmingdale, New York) and programmed to perform cycles of 60 min incubation at 32°C followed by a 30 s pulse of sonication set at 80% power. Samples were incubated, without shaking, and immersed in the water of the sonicator bath. After a round of 24 cycles, a 10 µl aliquot of the amplified material was diluted into 90 µl of normal transgenic mouse brain homogenate and a new round of 24 PMCA cycles was performed. This procedure was repeated four times to reach a final 10^6^-fold dilution of the initial sCJD brain homogenate.

### Sucrose gradient ultracentrifugation

The 400 µl aliquots of 10% brain homogenate in PBS, pH 7.4, containing 2% Sarkosyl were clarified by centrifugation at 500×g for 5 min and carefully layered onto the top of the 10–45% sucrose gradient. The sucrose gradient was prepared in PBS, pH 7.4, containing 1% Sarkosyl in Thinwall Polyallomer (13×51 mm) tubes (Beckman, Palo Alto, California). The ultracentrifugation was performed at 50,000 rpm for 73 min at 5°C in Optima TL ultracentrifuge (Beckman, Palo Alto, California) equipped with Beckman SW 55 Ti rotor. These conditions correspond to the adjusted proportionality constant k = 58.7 and angular velocity ω = 5236 rad/s. Observed sedimentation coefficients s_obs_ were calculated from formula s_obs_ = k/(ω^2^twhere t is the centrifugation time. The S_20,w_ values for given angular velocity, prion particle density 1.35 g/ml [61], and sucrose density and viscosity were calculated as described [62], [63]. The second approach to estimate the S values for the upper layers of sucrose gradient was calibration with bovine serum albumin (BSA, S = 4.4, MW = 67 kDa), alcohol dehydrogenase (ADH, S = 7.9, MW = 150 kDa), thyroglobulin monomer (TG, S = 12.0, MW = 335 kDa), and apoferitin (AF, S = 17.0, MW = 443 kDa) [63]. After the centrifugation, the 200 or 400 µl fractions of gradients were collected from the bottom of the tube.

### Statistical analysis

We investigated the effect of the following demographic and laboratory variables on survival: sex; age at onset; duration of the disease; electrophoretic Type of PrP 27-30; and the concentration and stability of PrP^Sc^ in Gdn HCl before and after PK treatment [22]. In the comparison of different groups, P values were calculated using Anova. Cumulative survival curves were constructed by the Kaplan–Meier method, both overall and by stratifying for each of the above variables. Comparisons of survival curves among groups were carried out by the log rank (Mantel-Cox) and generalized Wilcoxon test. All the statistical analyses were performed using SPSS 19 software (SPSS Inc., Chicago, Illinois).

## Supporting Information

Figure S1 A typical amplification of sCJD prions in sPMCA using brain homogenate of Tg mice expressing human PrP^C^(129M) and with QuIC using recombinant human PrP(23-231, 129M) substrate. (**A**) Sonication-driven sPMCA with brain PrP^C^ substrate preserves the differences in mobility of unglycosylated PrP^Sc^ in three MM1 and three MM2 sCJD PrP^Sc^ after four rounds of amplification and final dilution of brain sCJD prions 10^6^-fold. Asterisk signifies residual full length PrP after PK treatment. (**B**) 16 kDa protease-resistant fragments of rhuPrP^QuIC^ detected after QuIC by WBs developed with monoclonal antibody 12B2 (epitope residues 89–93) (21) or 3F4 (epitope residues 107–112) (9). The sCJD seeds were in QuIC reaction diluted 10^4^-fold.
(TIF)

Figure S2 The time course of QuIC reaction and comparison with sPMCA. (**A**) The typical results of QuIC seeded with different sCJD prions and low background levels of *de novo* PrP. (**B**) The amplification of sCJD PrP^Sc^ after four rounds of sPMCA (n = 20) correlates to a highly significant degree with QuIC (n = 20). The amplification index is the ratio between the concentration of PrP^Sc^ before and after PMCA measured with CDI. The data points are averages of three PMCA experiments, each measured in triplicate with CDI.
(TIF)

Figure S3 Continuum of amplification indexes of PrP^Sc^ recorded with both sPMCA and QuIC in MM1 and MM2 sCJD. (**A**) Amplification index obtained with sPMCA for (red circles) MM1 (n = 10) and (blue squares) MM2 (n = 10) sCJD cases. (**B**) Amplification index obtained with QuIC for (red circles) MM1 (n = 10) and MM2 (n = 10) sCJD. In both sPMCA and QuIC, the differences observed between MM1 and MM2 samples are not statistically significant. The amplification index was determined as described in legend for **Figure 1** and the data points are averages ± SEM obtained from three independent PMCA experiments, each measured in triplicate with CDI.
(TIF)

Figure S4 The CDI demonstrated the same end point concentrations of recombinant PrP substrate in samples with PK treated or untreated seeds at the end of the QuIC reaction. The total (substrate+seed) PrP concentration was measured with CDI in duplicate in six samples at the end of QuIC reaction.
(TIF)

Figure S5 Calibration of sucrose gradient ultracentrifugation with standard proteins. The (**A**) elution profile and (**B**) calibration plot for estimating S_20_,_w_ and mass. The calibrants were bovine serum albumin (BSA, S = 4.4, MW = 67 kDa), alcohol dehydrogenase (ADH, S = 7.9, MW = 150 kDa), thyroglobulin monomer (TG, S = 12.0, MW = 335 kDa), and apoferitin (AF, S = 17.0, MW = 443 kDa) (20). The protein content in each fraction was determined with BCA protein assay (Pierce, Rockford, Illinois.).
(TIF)

Figure S6 Sucrose gradient ultracentrifugation of control samples containing only PrP^C^. The (**A**) PrP^C^ in the human platelets isolated from blood of healthy donors and (**B**) in a control brain of the patient with other than prion disease (OND). The distribution of PrP^C^ (green bars), total PrP^Sc^ (blue bars), and rPrP^Sc^ (red bars) in sucrose fractions was determined with and without PK treatment by CDI. The PrP^Sc^ and rPrP^Sc^ values oscillating around zero were used to establish the cutoff and baseline sensitivity limit of CDI in each fraction. The bars represent average ± SEM from CDI performed in triplicate.
(TIF)

Figure S7 Fractionation by ultracentrifugation in sucrose gradient and protease sensitivity of samples taken from frontal cortex of individual (left column) MM1 (n = 6) and (right column) MM2 sCJD (n = 6) sCJD cases. The distribution of PrP^C^ (green bars), total PrP^Sc^ (blue bars), and rPrP^Sc^ (red bars) in sucrose fractions was determined with and without PK treatment by CDI. The bars represent average ± SEM from CDI performed in triplicate.
(TIF)

Table S1 Descriptive statistics of the data and demographics of sCJD cases.
(DOC)
