# Supplementary figures and images for: Small Protease Sensitive Oligomers of PrPSc in Distinct Human Prions Determine Conversion Rate of PrPC
Source: PLoS Pathog. 2012 Aug 2;8(8):e1002835. doi: 10.1371/journal.ppat.1002835 (PMC3410855; doi:10.1371/journal.ppat.1002835)

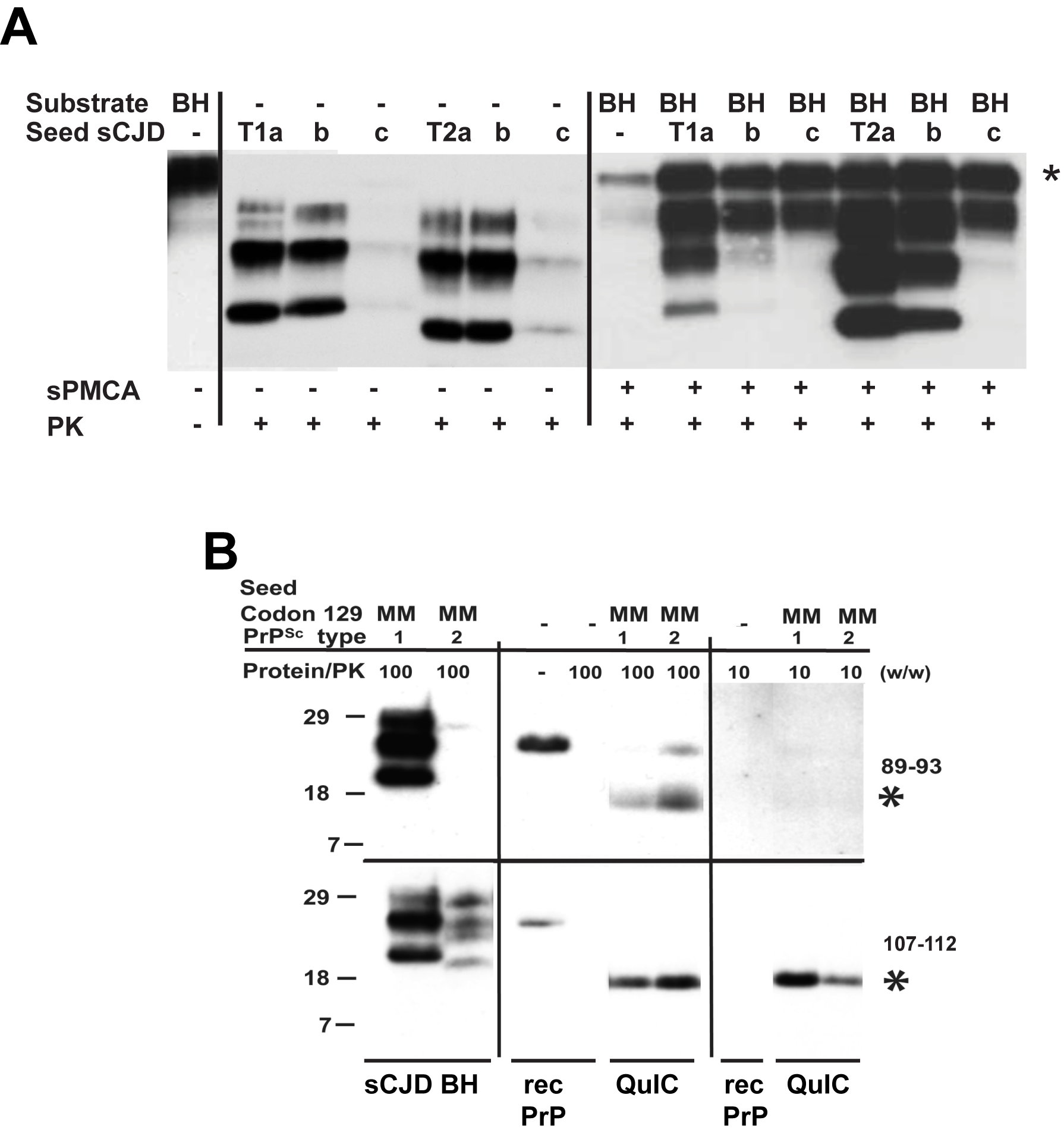

Supplement: Figure S1 — A typical amplification of sCJD prions in sPMCA using brain homogenate of Tg mice expressing human PrPC(129M) and with QuIC using recombinant human PrP(23-231, 129M) substrate. (A) Sonication-driven sPMCA with brain PrPC substrate preserves the differences in mobility of unglycosylated PrPSc in three MM1 and three MM2 sCJD PrPSc after four rounds of amplification and final dilution of brain sCJD prions 106-fold. Asterisk signifies residual full length PrP after PK treatment. (B) 16 kDa protease-resistant fragments of rhuPrPQuIC detected after QuIC by WBs developed with monoclonal antibody 12B2 (epitope residues 89–93) (21) or 3F4 (epitope residues 107–112) (9). The sCJD seeds were in QuIC reaction diluted 104-fold. (TIF) [file ppat.1002835.s001.tif]

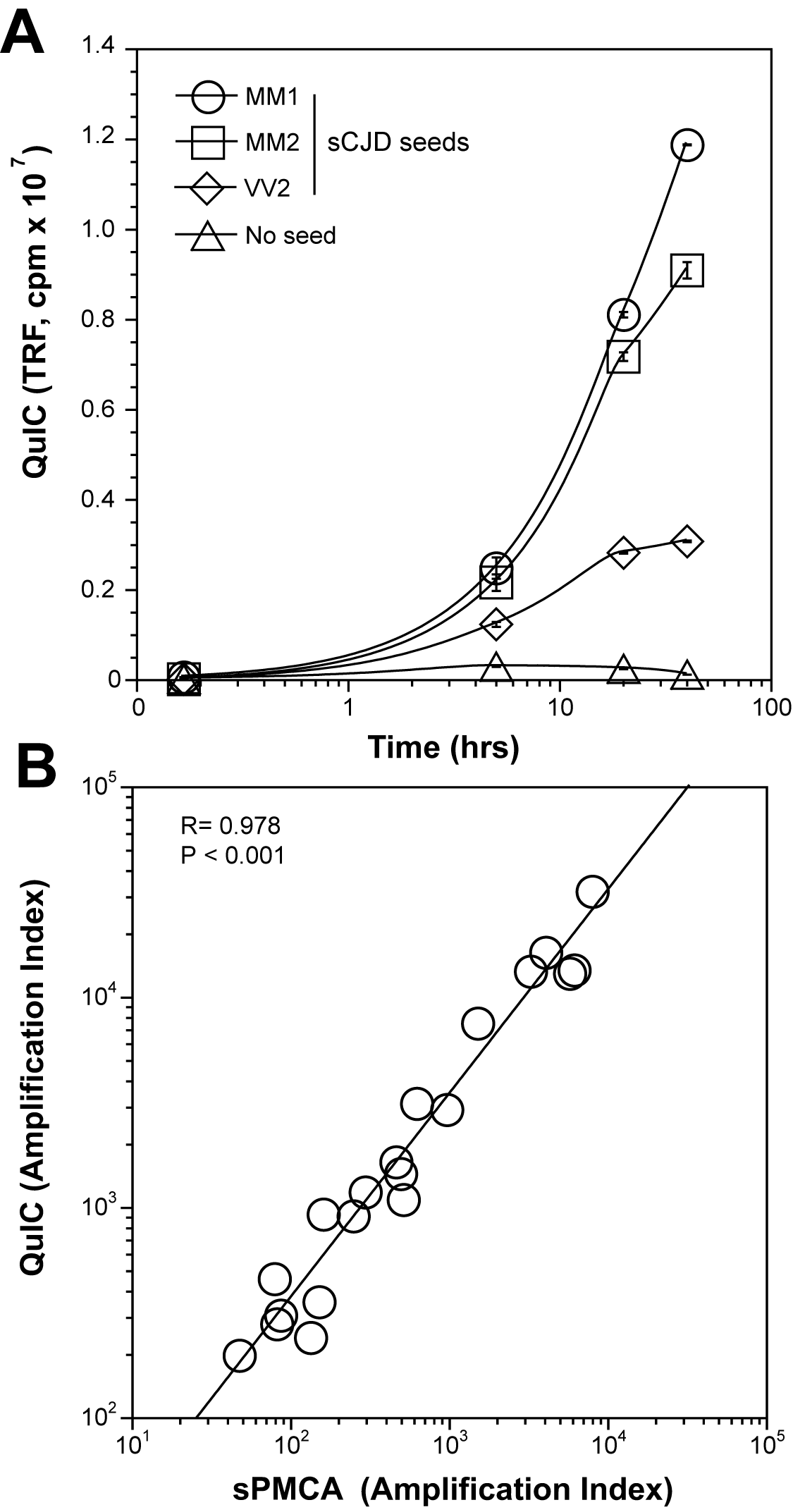

Supplement: Figure S2 — The time course of QuIC reaction and comparison with sPMCA. (A) The typical results of QuIC seeded with different sCJD prions and low background levels of de novo PrP. (B) The amplification of sCJD PrPSc after four rounds of sPMCA (n = 20) correlates to a highly significant degree with QuIC (n = 20). The amplification index is the ratio between the concentration of PrPSc before and after PMCA measured with CDI. The data points are averages of three PMCA experiments, each measured in triplicate with CDI. (TIF) [file ppat.1002835.s002.tif]

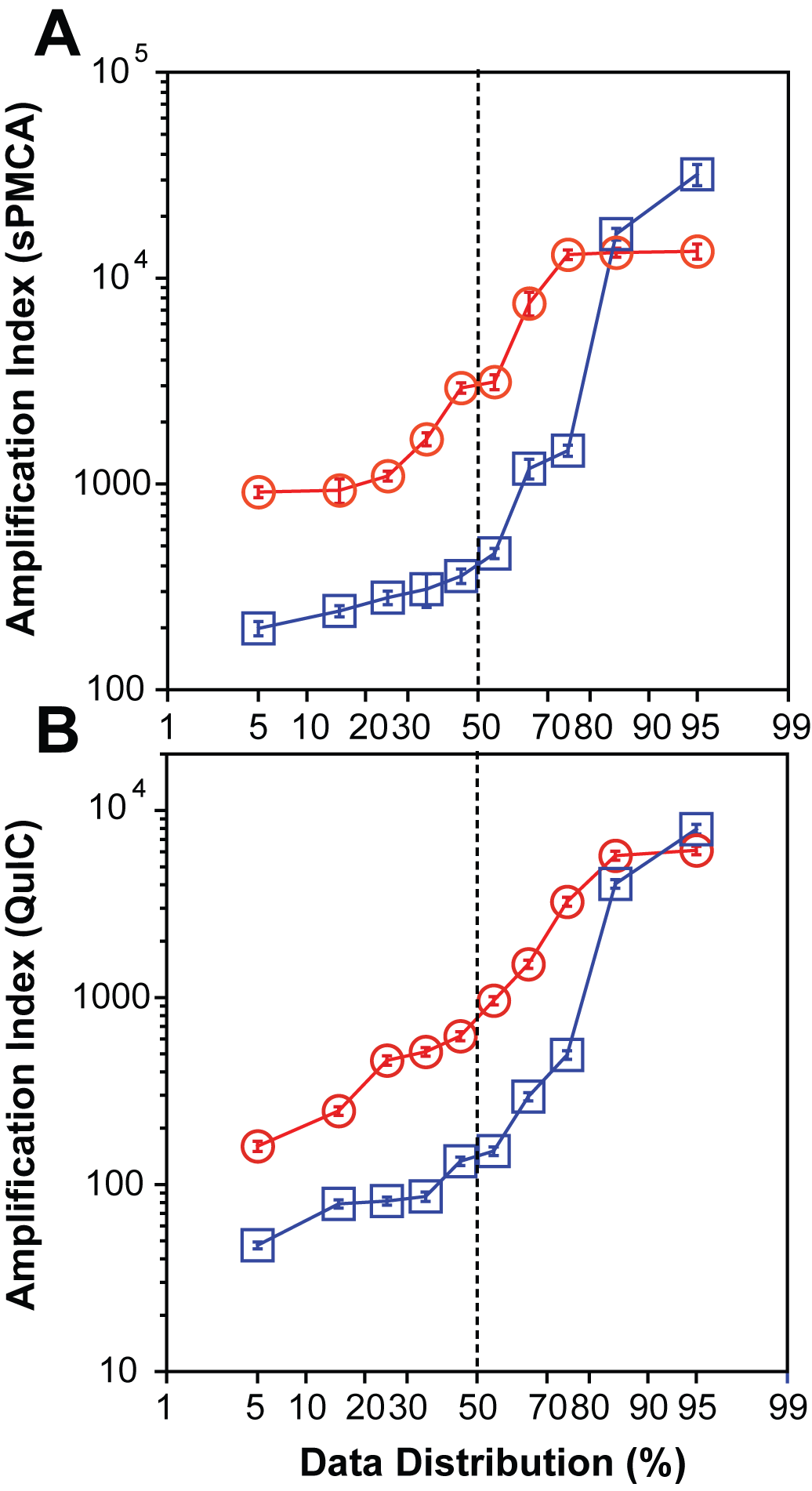

Supplement: Figure S3 — Continuum of amplification indexes of PrPSc recorded with both sPMCA and QuIC in MM1 and MM2 sCJD. (A) Amplification index obtained with sPMCA for (red circles) MM1 (n = 10) and (blue squares) MM2 (n = 10) sCJD cases. (B) Amplification index obtained with QuIC for (red circles) MM1 (n = 10) and MM2 (n = 10) sCJD. In both sPMCA and QuIC, the differences observed between MM1 and MM2 samples are not statistically significant. The amplification index was determined as described in legend for Figure 1 and the data points are averages ± SEM obtained from three independent PMCA experiments, each measured in triplicate with CDI. (TIF) [file ppat.1002835.s003.tif]

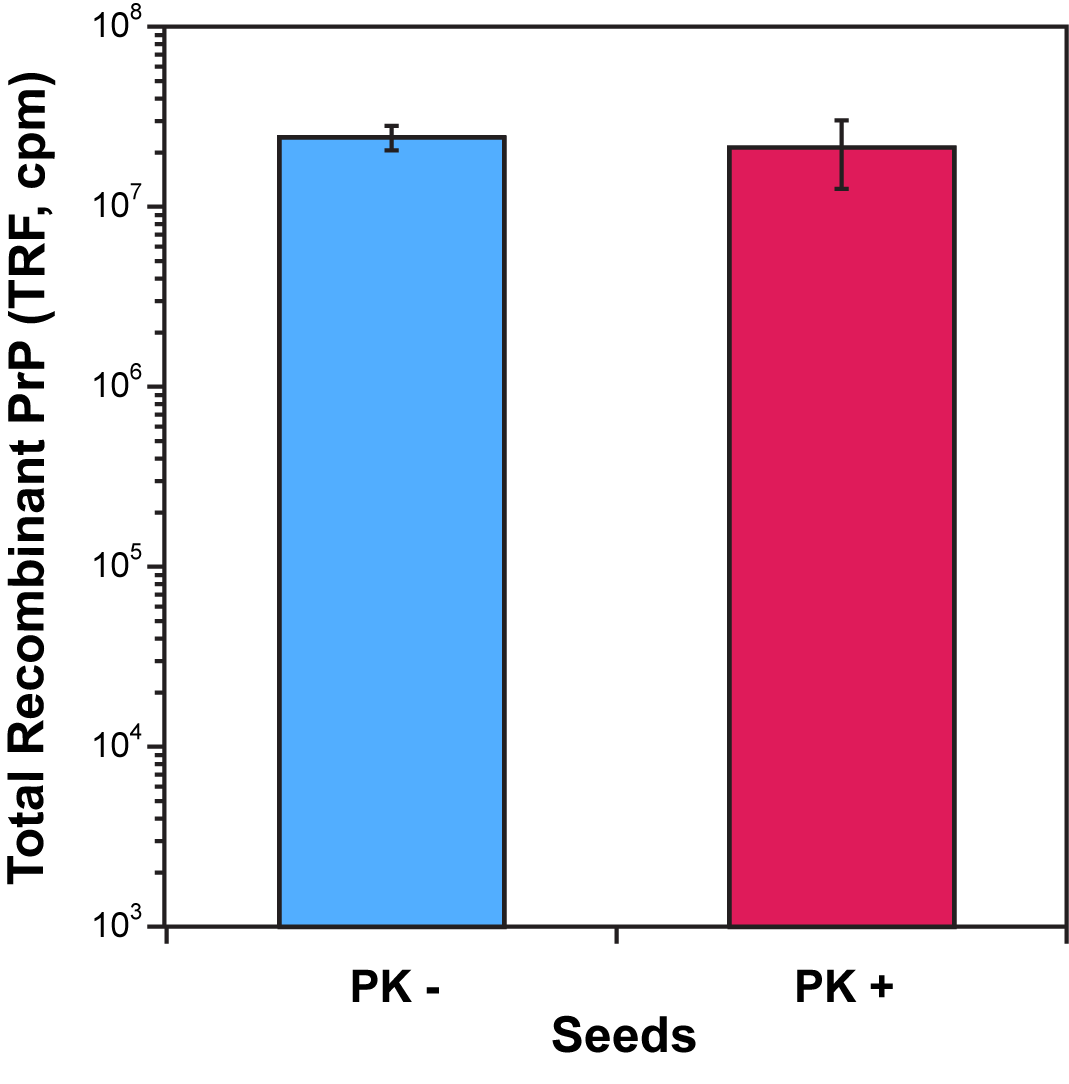

Supplement: Figure S4 — The CDI demonstrated the same end point concentrations of recombinant PrP substrate in samples with PK treated or untreated seeds at the end of the QuIC reaction. The total (substrate+seed) PrP concentration was measured with CDI in duplicate in six samples at the end of QuIC reaction. (TIF) [file ppat.1002835.s004.tif]

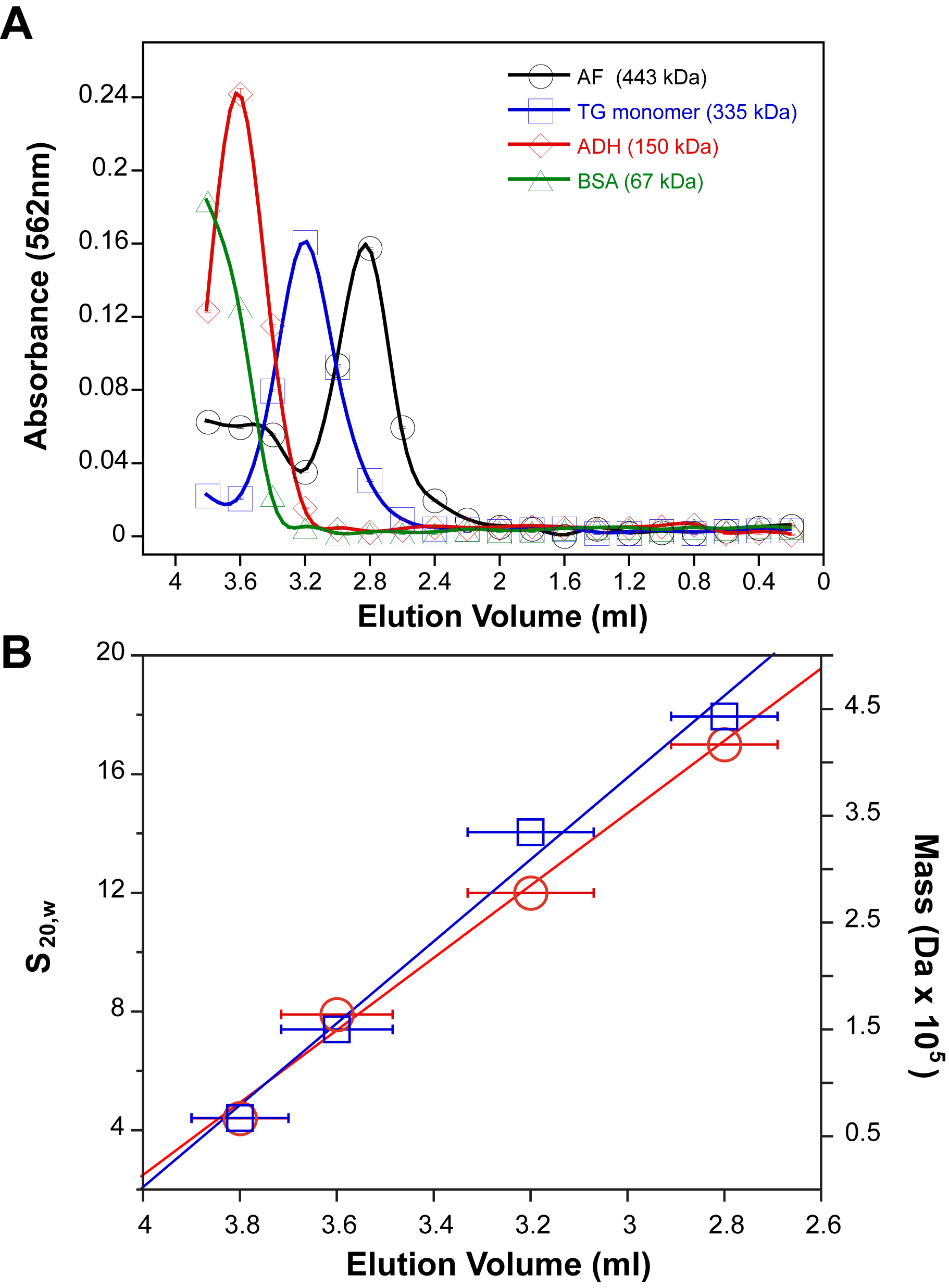

Supplement: Figure S5 — Calibration of sucrose gradient ultracentrifugation with standard proteins. The (A) elution profile and (B) calibration plot for estimating S20,w and mass. The calibrants were bovine serum albumin (BSA, S = 4.4, MW = 67 kDa), alcohol dehydrogenase (ADH, S = 7.9, MW = 150 kDa), thyroglobulin monomer (TG, S = 12.0, MW = 335 kDa), and apoferitin (AF, S = 17.0, MW = 443 kDa) (20). The protein content in each fraction was determined with BCA protein assay (Pierce, Rockford, Illinois.). (TIF) [file ppat.1002835.s005.tif]

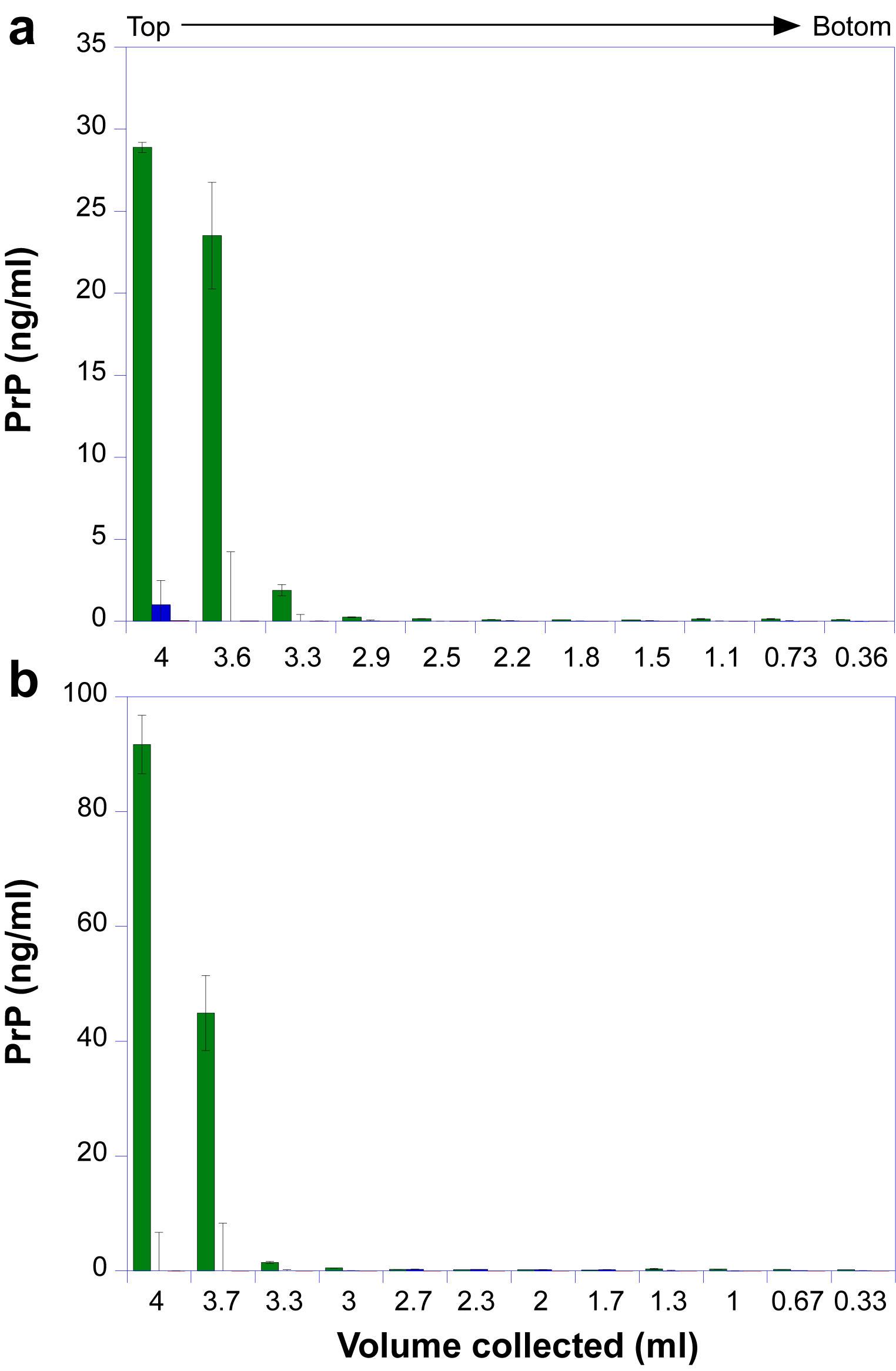

Supplement: Figure S6 — Sucrose gradient ultracentrifugation of control samples containing only PrPC. The (A) PrPC in the human platelets isolated from blood of healthy donors and (B) in a control brain of the patient with other than prion disease (OND). The distribution of PrPC (green bars), total PrPSc (blue bars), and rPrPSc (red bars) in sucrose fractions was determined with and without PK treatment by CDI. The PrPSc and rPrPSc values oscillating around zero were used to establish the cutoff and baseline sensitivity limit of CDI in each fraction. The bars represent average ± SEM from CDI performed in triplicate. (TIF) [file ppat.1002835.s006.tif]

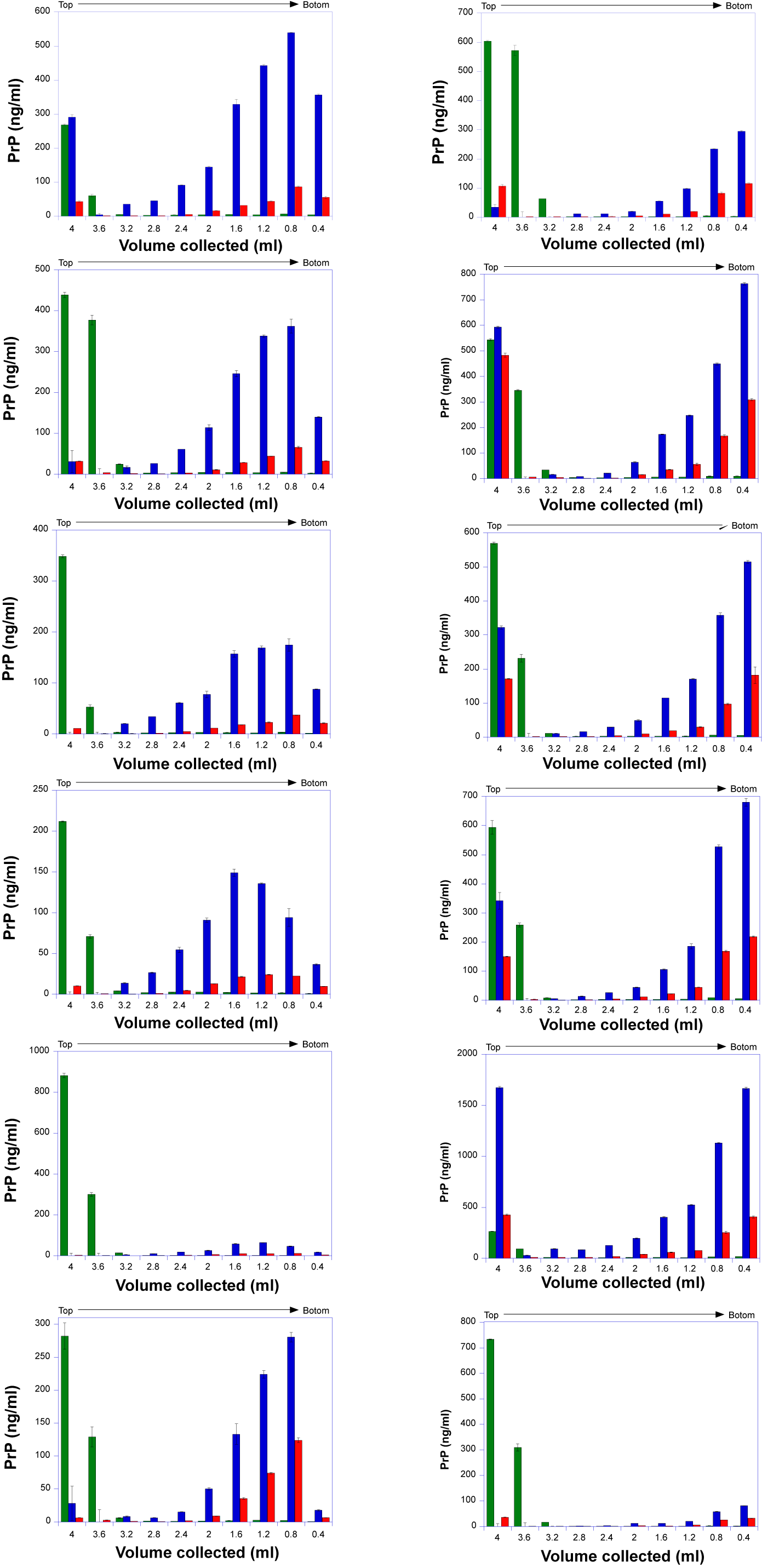

Supplement: Figure S7 — Fractionation by ultracentrifugation in sucrose gradient and protease sensitivity of samples taken from frontal cortex of individual (left column) MM1 (n = 6) and (right column) MM2 sCJD (n = 6) sCJD cases. The distribution of PrPC (green bars), total PrPSc (blue bars), and rPrPSc (red bars) in sucrose fractions was determined with and without PK treatment by CDI. The bars represent average ± SEM from CDI performed in triplicate. (TIF) [file ppat.1002835.s007.tif]
