# Supplementary material for: Small Protease Sensitive Oligomers of PrPSc in Distinct Human Prions Determine Conversion Rate of PrPC
Source: PLoS Pathog. 2012 Aug 2;8(8):e1002835. doi: 10.1371/journal.ppat.1002835 (PMC3410855; doi:10.1371/journal.ppat.1002835)
Supplement: Table S1 — Descriptive statistics of the data and demographics of sCJD cases. (DOC) [file ppat.1002835.s008.doc]

**Supplemental Table 1**. Descriptive statistics of the data and demographics of sCJD cases.

| **PRNP**  **codon 129** |  | **MM** | | | | | | | | | | | | |
| --- | --- | --- | --- | --- | --- | --- | --- | --- | --- | --- | --- | --- | --- | --- |
| **WB type** |  | **1** | | | | | |  | **2** | | | | | |
| **Variable** | **Units** | **n** | **Minimum** | **Maximum** | **Mean** | **±** | **S.E.M.** | **Sig** | **n** | **Minimum** | **Maximum** | **Mean** | **±** | **S.E.M.** |
| **Sex** | F/M | 3/7 |  |  |  |  |  | NS | 5/5 |  |  |  |  |  |
| **Age** | years | 10 | 54 | 74 | 64.7 | ± | 1.8 | 0.003 | 10 | 60 | 92 | 78.8 | ± | 3.4 |
| **Disease**  **Duration** | month | 10 | 1.4 | 5.4 | 3.1 | ± | 0.4 | <0.001 | 10 | 3.1 | 20.1 | 12.1 | ± | 1.8 |
| **PrP^Sc^** | ng/ml | 10 | 22 | 547 | 189 | ± | 57.9 | 0.004 | 10 | 28 | 2996 | 1184 | ± | 296 |
| **rPrP^Sc^** | ng/ml | 10 | 12 | 234 | 92 | ± | 26.9 | 0.006 | 10 | 4 | 1181 | 501 | ± | 127 |
| **sPrP^Sc^** | ng/ml | 10 | 11 | 313 | 97 | ± | 31.8 | 0.004 | 10 | 24 | 1815 | 684 | ± | 175 |
| **PrP^Sc^**  **Gdn HCl_1/2_** | M | 10 | 2.34 | 3.04 | 2.77 | ± | 0.08 | 0.010 | 10 | 2.58 | 3.51 | 3.11 | ± | 0.09 |
| **rPrP^Sc^**  **Gdn HCl_1/2_** | M | 10 | 2.95 | 3.34 | 3.12 | ± | 0.05 | <0.001 | 10 | 2.61 | 2.89 | 2.71 | ± | 0.03 |
| **PK-Induced**  **Change in**  **Stability** | **Δ** M | 10 | 0.07 | 0.91 | 0.36 | ± | 0.09 | <0.001 | 10 | -0.80 | 0.05 | -0.40 | ± | 0.09 |
|  | **Δ** Fapp | 10 | 0.13 | 0.72 | 0.34 | ± | 0.06 | <0.001 | 10 | -0.65 | 0.25 | -0.23 | ± | 0.07 |
